# Anti-PD-1 Therapy-Associated Perforating Colitis

**DOI:** 10.1155/2018/3406437

**Published:** 2018-05-31

**Authors:** Romulo Celli, Harriet M. Kluger, Xuchen Zhang

**Affiliations:** ^1^Department of Pathology, Section of Medical Oncology, Yale University School of Medicine, New Haven, CT, USA; ^2^Department of Medicine, Section of Medical Oncology, Yale University School of Medicine, New Haven, CT, USA

## Abstract

Inhibition of immune checkpoint T cell regulatory molecules by using programmed cell death protein 1 (PD-1), or its ligand (PDL-1), and cytotoxic T-lymphocyte-associated antigen 4 (CTLA-4) has been increasingly used to treat advanced malignancies. The immune-related adverse effects associated with these treatments such as diarrhea, colitis, and CTLA-4 treatment-associated perforating colitis have been reported. However, anti-PD-1/PD-L1-associated perforating colitis has rarely been reported. We report a case of colonic perforation in a patient recently treated with pembrolizumab, a PD-1 inhibitor for metastatic melanoma. Awareness of anti-PD-1/PD-L1-associated colitis and perforation will facilitate a timely diagnosis and management as they are increasingly used in oncology.

## 1. Introduction

Inhibition of the immune checkpoint T cell regulatory molecules has become an increasingly used oncologic strategy in several advanced malignancies, including metastatic melanoma [1]. Monoclonal antibodies designed against molecules such as programmed cell death protein 1 (PD-1), or its ligand (PDL-1), and cytotoxic T-lymphocyte-associated antigen 4 (CTLA-4) comprise the most commonly used agents. Previous reports of the immune-related adverse effects associated with these treatments abound [2, 3]. One common complication of these treatments is diarrhea. A subset of these show radiologically identifiable inflammatory changes of the colon. Diarrhea is seen more commonly with CTLA-4 treatment [4], and several cases of severe colitis with colonic perforation have been reported [5–7]. Diarrhea and colitis have been reported much less frequently in the setting of PD-1 inhibitor therapy [8, 9]. Herein, we report a case of colonic perforation in a patient undergoing recent treatment with pembrolizumab, a PD-1 inhibitor for metastatic melanoma.

## 2. Case Report

**Figure 1:**
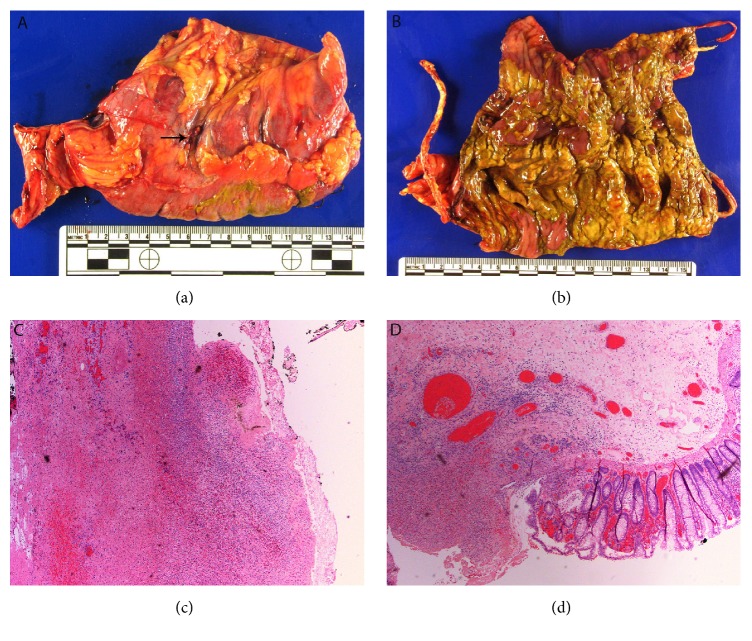
(a) perforation (arrow). (b) Colonic mucosa with yellowish exudate and multifocal ulcers. (c) Transmural necrosis (H&E). (d) Ulcer and adjacent colonic mucosa.

A 51-year-old woman presented with fatigue, nausea, and vomiting for three days. She was diagnosed with metastatic melanoma to pelvic nodes in July 2014 and was treated with ipilimumab (anti-CTLA-4) in combination with nivolumab (PD-1 inhibitor) between September 2014 and April 2015, receiving a total of 4 cycles of combination therapy and 9 cycles of nivolumab monotherapy with an initial mixed response followed by slow progression of disease. In April 2015 she was enrolled on a study of radiation (to pelvic mass) in combination with pembrolizumab (PD-1 inhibitor), receiving a total of 9 cycles of pembrolizumab with no toxicities. In December 2016 she was found to have new brain metastases and in January 2017 pembrolizumab was added. The day following her second dose of pembrolizumab, she developed fatigue and nausea and began having intermittent vomiting and diarrhea. Abdominal CT scan demonstrated diffuse colitis. Infectious studies including* C. difficile* antigen, stool culture, viral PCR, and ova and parasites exam were all negative. She was started on methylprednisolone 2m/kg/day. Over four days of hospitalization, her abdominal pain worsened and she developed melena, which progressed to bright red blood per rectum. She was given infliximab at 10mg/kg. Repeat imaging performed 48 hours later due to severe abdominal distension showed large amounts of free air with gaseous distention of large and small bowel loops, consistent with perforation in the context of colitis with ileus. She was taken to the operating room for emergent bowel resection and a perforation site was identified at the transverse colon. The resected transverse colon serosa was congested and dusky with site of perforation identified (Figure 1(a)). The colonic mucosa revealed diffusely edematous folds as well as confluent areas of yellowish exudate and multifocal ulcers (Figure 1(b)). Histologic sections confirmed the presence of transmural necroinflammation and multifocal ulceration (Figures 1(c) and 1(d)). The findings were of a fulminant colitis with multifocal ulceration and perforation. No evidence of metastatic melanoma to the bowel was identified.

## 3. Discussion

There is evidence of improved overall and progression-free survival from large-scale clinical trials in patients with metastatic melanoma treated with the immune checkpoint inhibitors [4, 10, 11]. The general mechanism of these treatments is blockade of T cell regulatory molecules, leading to potentiation of antitumor immune effects. Targeted monoclonal antibodies have been developed against PD-1 and its ligand (PDL-1), as well as against cytotoxic T-lymphocyte associated protein 4, CTLA-4. These have been found to be active against a number of tumor types. The PD-1 and PD-L1 inhibitors are currently indicated in cases of advanced/unresectable melanoma and certain cases of non-small cell lung cancer, among other cancer types. Known adverse effects of T cell activation by this mechanism are characterized as immune-related adverse effects (irAEs) and include injury to several potential organs. Among the most common irAEs are diarrhea (increased frequency in stools) and enterocolitis (abdominal pain and/or imaging findings consistent with inflamed bowel). As with most organ-specific irAEs, CTLA-4 inhibitors tend to have an increased rate compared to the anti-PD1/PDL-1 agents. The incidence of diarrhea in patients taking CTLA-4 inhibitors has been about 30%, and about 10% of these patients develop severe colitis [4]. On the other hand, diarrhea has complicated anti-PD-1 treatment in approximately 16% of cases and severe diarrhea has been seen in only about 1-3% of patients [8, 9]. Prior cases of CTLA-4 inhibitor therapy-associated colitis with perforation have been reported [5–7]. To our knowledge, only one case of intestinal perforation due to anti-PD-1 therapy has been recently described[12].

The pathologic correlations to PD-1 inhibitor associated colitis were recently described by Chen et al. in a published series of eight cases [13]. The majority of these (five of eight) showed histologic acute colitis with crypt abscesses and frequent epithelial apoptotic bodies. The remaining cases demonstrated findings indistinguishable from lymphocytic colitis. Patients with recurrent bouts of colitis developed chronic colitis-type histologic findings including lamina propria expanded by inflammation, basal lymphoplasmacytosis, and mild crypt architectural abnormalities. Our patient did not have a history of recurrent colitis. Also, the remaining areas of intact mucosa showed mild crypt architectural distortion and scattered epithelial apoptotic bodies. These findings are consistent with injury of subacute to acute duration and are consistent with the reported effects of mild chronic inflammatory changes in the setting of anti-PD-1 therapy. Whether or not the patient's prior ipilimumab therapy contributed to colonic mucosal injury is difficult to assess; however, she had 11 cumulative doses of pembrolizumab, which would likely be enough to account for the changes seen. Implicating ipilimumab in this pathologic process would be speculative at best. The focal areas of intense acute colitis, ulceration, and perforation seen in our case were not reported in the series by Chen et al. [13]. However, given the biologic mechanism of action, it is not unreasonable to think that these agents could produce such a dramatic clinical and histologic picture.

Our patient developed fulminant colitis with multifocal ulceration and perforation following recent two doses of pembrolizumab. Although treatment with high dose steroids was initiated upon presentation to the hospital, the colitis progressed and did not respond to infliximab. Intestinal perforation should be considered a potential complication, though rare, of any immune checkpoint inhibitor therapy.

## Data Availability

The data used to support the findings of this study are included within the article.
